# Sensory Recovery Outcome after Digital Nerve Repair in Relation to Different Reconstructive Techniques: Meta-Analysis and Systematic Review

**DOI:** 10.1155/2013/704589

**Published:** 2013-07-30

**Authors:** Felix J. Paprottka, Petra Wolf, Yves Harder, Yasmin Kern, Philipp M. Paprottka, Hans-Günther Machens, Jörn A. Lohmeyer

**Affiliations:** ^1^Department of Plastic Surgery and Hand Surgery, Klinikum rechts der Isar, Technische Universität München, Ismaninger Straße 22, 81675 Munich, Germany; ^2^Institut für Medizinische Statistik und Epidemiologie, Medizinische Biometrie, Klinikum rechts der Isar, Technische Universität München, Ismaninger Straße 22, 81675 Munich, Germany; ^3^Institut für Klinische Radiologie, Campus Grosshadern, Ludwig-Maximilians-Universität München, Marchioninistraße 15, 81377 München, Germany

## Abstract

Good clinical outcome after digital nerve repair is highly relevant for proper hand function and has a significant socioeconomic impact. However, level of evidence for competing surgical techniques is low. The aim is to summarize and compare the outcomes of digital nerve repair with different methods (end-to-end and end-to-side coaptations, nerve grafts, artificial conduit-, vein-, muscle, and muscle-in-vein reconstructions, and replantations) to provide an aid for choosing an individual technique of nerve reconstruction and to create reference values of standard repair for nonrandomized clinical studies. 87 publications including 2,997 nerve repairs were suitable for a precise evaluation. For digital nerve repairs there was practically no particular technique superior to another. Only end-to-side coaptation had an inferior two-point discrimination in comparison to end-to-end coaptation or nerve grafting. Furthermore, this meta-analysis showed that youth was associated with an improved sensory recovery outcome in patients who underwent digital replantation. For end-to-end coaptations, recent publications had significantly better sensory recovery outcomes than older ones. Given minor differences in outcome, the main criteria in choosing an adequate surgical technique should be gap length and donor site morbidity caused by graft material harvesting. Our clinical experience was used to provide a decision tree for digital nerve repair.

## 1. Introduction

Nerve injuries are common in trauma surgery and appear more often if the upper extremity is affected [1]. In about 10% of all hand injuries, nerves, which require surgical treatment, are involved [2]. As a result, numbness and impairment of motor function may occur [3]. After performed nerve repair, intensive and time-consuming rehabilitation is needed. The highest incidence of nerve injuries can be observed in young men aged 16–35, with women only contributing to 20–30% of all cases [1, 2]. The most frequently damaged nerves associated with injuries of the upper extremity are the common and proper digital nerves, followed by the median and ulnar nerves [2]. Sick leave and sometimes the need for change in profession as well as partial or even permanent total disability may have a severe economic impact on the patient and society [4, 5]. 

Thus, digital nerve lesions require surgical revision. Within this paper we focused on the major surgical techniques for digital nerve repair. 

End-to-end nerve coaptations (synonym: direct coaptation) and nerve grafting have been used preferentially to repair severed nerves for a long time now. After introduction of the surgical microscope into daily clinical use in the early 1960s [6], nerve repair became more accurate and results improved. Every nerve repair should be performed under adequate magnification usually implying the surgical microscope. 

End-to-side nerve repair (synonym: terminolateral coaptation) is a procedure in which an injured nerve ending is coapted to the side of a functioning donor nerve nearby. An epineural window serves as a connection point between the two nerves, while the extent of the perineural fenestration remains controversial [7]. The end-to-side nerve coaptation can be used to repair severed digital nerves, mostly caused by previous hand injuries, when direct tension-free coaptation is not possible [8–12].

The autologous nerve graft is the gold standard for nerve injuries that cannot be repaired by direct tension-free coaptation [12, 13]. The most common source of autologous nerve grafts is the sural nerve. This autologous graft is quite easily harvested and almost always has an appropriate diameter for digital nerve reconstruction. Other common sources of nerve grafts are posterior interosseous nerve and medial antebrachial cutaneous nerve. However, the use of autologous nerve grafts carries the risk of donor site morbidity including sensory loss within the area of harvest and the corresponding peripheral nerve field, painful neuroma, and scar formation. Increased operating time and limited harvest sites are also disadvantages. 

Artificial conduits have become an alternative to the use of nerve grafts in order to bridge a limited nerve defect, which cannot be tension-free coapted. The artificial conduit grafts are mostly composed of collagen and/or polyglycolic acid (PGA) [13]. Artificial conduits do not cause donor site morbidity. Surgeons may also be more willing to properly debride nerve endings when conduit interposition substitutes the need for direct coaptation [14]. The artificial conduit is hollow inside, thus behaving like a guiding growth chamber. Furthermore, the artificial conduit seems to have a protective function by preventing neuroma formation and the ingrowth of fibrous tissue [15]. Eventually, the artificial nerve guide degrades and is resorbed after it has accomplished its work [16]. It is recommended to use artificial conduits only up to a maximum of 3 cm nerve defect length in order to achieve acceptable sensory recovery [14]. 

Furthermore, vein reconstructions can also be used to bridge a nerve gap, which cannot be approximated without any tension. For digital nerve repair, veins can be harvested, for example, from the ipsilateral dorsum of the hand or the palmar forearm. The use of vein conduits is also associated with risks such as donor site requirement and scar formation around the collapsing vein, which may hinder appropriate regeneration over extended distances [17].

Accordingly, muscle-in-vein reconstruction can be an alternative to prevent collapse of the vein graft. In this case, fresh skeletal muscle tissue is placed inside the vein conduit in order to keep the vein's inner space open, offer the sprouting axons a collagen/laminin axis to grow in, and let the graft become more flexible. Sanes et al. demonstrated that the histological resemblance of muscle and nerve basement membrane further promotes hope for advanced functional regeneration [18]. Also, muscle alone can be interposed into a nerve defect to allow regeneration [19]. The muscle graft provides a clinically acceptable graft material, which is abundantly available [19]. Nevertheless, dispersion of regenerating axons out of the muscle is a known problem [20, 21].

For digital replantation, methods like direct nerve repair and nerve grafting play an important role. Remarkable improvements of microvascular surgical techniques have been the basis in order to successfully perform digital replantation. As survival rates for replanted fingers have become fairly impressive (up to 90%) [22], a better sensory and functional recovery should be the next main focus.

Finally, processed nerve allografts are now commercially available as Avance by AxoGen Inc. with a quite promising sensory recovery after treatment [23]. Those nerve allografts provide decellularized and predegenerated human nerve tissue for the restoration of nerve continuity—maintaining a microenvironment conducive to axonal regeneration [23]. This study outcome is very promising, but acquiring consent for allogeneic transplantation might be an issue for some patients—despite strict production control, there still could be a minor risk of infectious disease transmission [24].

Even though most techniques are common practice, literature lacks clear prognostic statements with regard to different reconstructive methods. The absence of comparable facts hinders proper medical judgment and the assessment of new data concerning specific methods for nerve reconstruction. Also, despite a considerable number of publications partly addressing this issue, most of them do not focus on elaborate and comparative characterizations about sensory recovery after digital nerve repair. The last study to sum up current scientific publications was published by Glickman and Mackinnon [25] in 1990. Due to multiple influencing factors compared to stand-alone nerve repair, digital replantations were regarded as a separate group. In virtue of its high clinical incidence and exclusive sensory quality, digital nerve lesions present an excellent basis for comparability of different techniques and were therefore chosen as the ideal clinical model [3, 26].

The aim of this work is therefore threefold.

- To summarize and compare the outcome of digital nerve reconstruction with different techniques including end-to-end and end-to-side coaptations, nerve grafts, artificial conduit-, vein-, muscle, and muscle-in-vein-reconstructions. Digital replantations were regarded as a separate group.
- To create reference values of standard repair for non-randomized clinical studies and to discuss publications, which had an interesting and valuable content, but did not fit into the modified Highet classification system.
- To provide an aid for choosing an individual technique of nerve reconstruction by formulating a treatment recommendation.

## 2. Methods

A broad range of papers have been reviewed for this publication. All clinical data found on proper palmar digital nerves as well as common digital nerves were evaluated for their feasibility to be included in our analysis.

### 2.1. Search Strategy

During the research process (06/2010–01/2012) all the available literature was scanned to verify if it might fit into the modified Highet classification for comparison (Table 1). For research purposes, PubMed (http://www.ncbi.nlm.nih.gov/pubmed/) was employed as a specific scientific search engine to identify the useful literature. Our search criteria were “digital nerve,” “nerve graft AND hand,” “nerve reconstruction AND hand,” “nerve repair AND hand,” “digital nerve repair,” “nerve laceration AND Hand,” “nerve gap AND hand,” “nerve repair AND finger” or “nerve regeneration AND hand”. Furthermore, the option “related articles,” which is offered by PubMed, helped in finding additional valuable publications. Besides this, references reported in papers, which have been proven to be useful for this article, were scanned to find even more beneficial papers. Additionally, our university library was scanned for suitable doctoral research studies. Authors, publication date, kind of treatment, the number of all treated nerves, which could be tracked within the follow-up period, the patient's age, follow-up period, timing of repair, gap length of the nerve defect, sensory recovery, and additional information were collected. A fundamental work was already published by Glickman and Mackinnon in 1990 and served as historical background for our research [25].

### 2.2. Selection Criteria

Static two-point discrimination (s2PD) and in some cases moving two-point discrimination (m2PD) are the prevailing techniques to indicate sensory recovery after surgical digital nerve repair. Thus, two-point discrimination (2PD) created the basis for comparison of different therapies, including end-to-end and end-to-side coaptations, nerve grafts, artificial conduit-, vein-, muscle, and muscle-in-vein-reconstructions. Digital replantations, being complex injuries of the hand, were regarded as a separate group, not taking into consideration the technique of nerve repair. The reason for this is that—besides the performed nerve repair—also the vessel reconstruction has a major impact on the final sensory and motoric recovery outcome. Mackinnon and Dellon [27] modified a classification by Highet and Sanders [28], named the Highet classification, using either s2PD or m2PD to grade sensory recovery (Table 1)—this classification system served as basis for grading sensory recovery outcome after previously executed digital nerve repair. Adults and pediatric patients were included in the analysis. Only publications, which had at least one year of follow-up, were used for statistical evaluation and became part of this meta-analysis.

### 2.3. Data Extraction

Two authors searched for usable publications separately and assessed the suitable data individually. Subsequently, the collected data were matched. A third author resolved any discrepancies if the first two authors disagreed.

### 2.4. Statistical Analysis

The statistical software package R version 2.13.1 (R Foundation for Statistical Computing, Vienna, Austria) with functions *metaprop *(R package: meta [29]) and *rma* (R package: metafor [30]) was used for the statistical analysis. Pooled estimates of proportions with corresponding 95% confidence intervals were calculated based on the Freeman-Tukey double arcsine transformation [31]. The DerSimonian-Laird random effects method [32] was used to pool the transformed proportions. The *I* ^2^ statistic and its connected chi-square test for heterogeneity were calculated as a measure of heterogeneity of the combined study results [33].

For evaluating the source of heterogeneity within the different techniques as well as to test for differences between the particular techniques, a metaregression using a mixed effects model was calculated. Forest plots were created for each technique, showing individual study proportions with confidence intervals and the overall random-effects pooled proportion. All statistical analyses were done using a 0.05 level of significance.

## 3. Results

### 3.1. Study Selection

**Figure 1:**
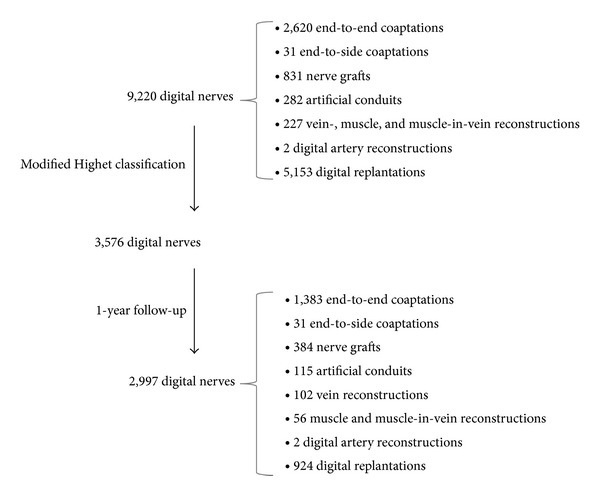
Selection criteria for digital nerve repair data. Selection criteria process for useful digital nerve repairs including their surgical techniques, which became part of the performed meta-analysis. Selection criteria for the 87 evaluated papers are the use of the modified Highet classification (Table 1) and an adequate follow-up period of at least one year.

During our research, 182 clinical papers were approved for further investigation. Among these, 87 were suitable for precise evaluation. Thus, overall 9,220 digital nerves were identified. From those, 3,576 eligible nerve repairs fulfilled defined criteria in order to compare sensory recovery using the modified Highet classification (Table 1). Due to a missing adequate follow-up of at least one year, another 579 repairs had to be excluded, finally ending up with 2,997 suitable nerves. A detailed overview concerning the selection process is shown in Figure 1. Those remaining 2,997 digital nerve repairs provided the basis for further analysis (Table 2 [3, 25, 34–48], Table 3 [8–12], Table 4 [3, 25, 36, 40, 43, 48–57], Table 5 [14, 58–64], Table 6 [36, 45, 56, 65–68], Table 7 [19, 37, 64, 69–71], and Table 8 [25, 72–77]). Some case reports, which were excluded from the meta-analysis to lower heterogeneity regarding the given sensory recovery outcomes, are nevertheless shown in Tables 2–8. Only studies that gave individual data were suitable to be included in our meta-analysis.

### 3.2. Sensory Recovery Outcome for Different Digital Nerve Repair Techniques

**Figure 2:**
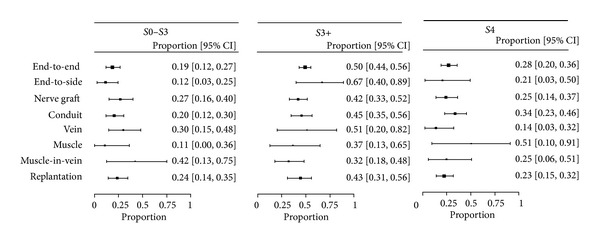
Overview of surgical techniques. Pooled estimates of proportions S0–3, S3+, and S4 with 95% confidence intervals from the random effects model for each technique (end-to-end and end-to-side coaptations, nerve grafts, artificial conduit-, vein-, muscle and muscle-in-vein reconstructions, and replantations). The proportions are drawn proportionally to the precision of the estimates.

The primary aim of the meta-analysis was to determine the overall proportion of patients with results S0–3, S3+, and S4 in comparison to other techniques. The pooled estimates are shown in Figure 2. Overall comparison of S0–3 results with S3+/S4 showed no significant superiority for a specific technique, but significant differences between the surgical techniques could be demonstrated by a detailed comparison of S0–3, S3+, and S4 (Table 9). Within the S3+ Highet classification, significantly higher sensory recovery rates could be identified for end-to-side coaptations compared to end-to-end coaptations or nerve grafting. Besides that, end-to-end coaptations or nerve grafts had slightly higher amounts of S4 results, meaning a more precise 2PD than end-to-side coaptations. All in all, sensory recovery outcomes of publications using the same technique for nerve reconstruction were quite heterogeneous (Tables 2–8).

### 3.3. Factors Affecting Sensory Recovery Outcome

**Figure 3:**
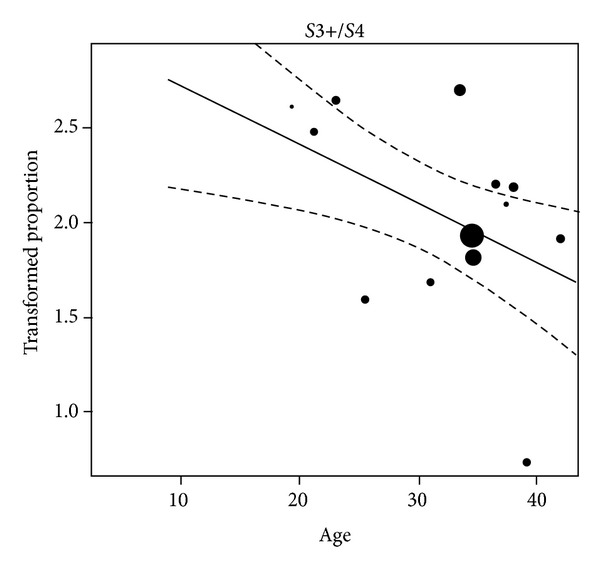
Metaregression: factor age/replantation. Freeman-Tukey double arcsine transformed proportions plotted against the mean age of the patients per study. The lines reflect the predicted effects with corresponding 95% confidence interval bounds. The transformed proportions are drawn proportionally to the inverse of the corresponding standard errors.

Based on the meta-regression, certain factors were identified to influence sensory recovery outcome. First of all, a comparison of publications focusing on end-to-end coaptations indicated that those culled from 1980 to date had a better sensory recovery outcome than those released from 1965 to 1979 (from 1965–1979: 66% S3/S4 (95% confidence interval: [45%–84%]), from 1980–today: 84% S3/S4 (95% confidence interval: [76%–92%])). The widespread clinical implementation of the surgical microscope started around 1965 [6]. For other surgical digital nerve repair techniques, no comparable correlation between publication date and sensory recovery outcome could be demonstrated. Comparison of the patient's age with sensory recovery outcome after one certain treatment also showed that after digital replantation younger patients had a significantly better outcome than older ones (Figure 3). No statistical significant evidence was found comparing the time of repair and follow-up with sensory recovery outcome. Finally, comparison of gap lengths up to 4 cm with sensory recovery outcome after artificial conduit or vein reconstructions did not reveal any significant difference.

## 4. Discussion

### 4.1. Comparison of Different Techniques with Regard to Sensory Recovery

So far, never before such a comprehensive patient collective has been analyzed with focus on sensory recovery outcome after performing certain digital nerve repair techniques. In different publications, which included all in all 2,997 digital nerves, a broad heterogeneity concerning sensory recovery could be shown (Tables 2–8). For digital nerve repair, there was practically no particular surgical technique superior to another. Only end-to-side coaptation seemed to have an inferior 2PD compared to end-to-end coaptation or nerve grafting—meaning that in subgroups S3+ and S4, end-to-side coaptations had significantly more S3+ results in comparison to end-to-end coaptations or nerve grafts. Also, end-to-end coaptations or nerve grafts had slightly higher amounts of S4 results, meaning a more precise 2PD than end-to-side coaptations. This may be the case, since histologically after end-to-side coaptation axons sprout out sideways, most likely resulting in a lower axon count in the regenerating nerve if compared to end-to-end coaptation. But taking everything into account, sensory recovery outcome after end-to-side coaptations was still quite useful with only 12% S0–3 results. Furthermore, after performed end-to-end coaptations newer publications demonstrated significantly better sensory recovery outcomes than older ones.

Additionally, this meta-analysis showed that youth was associated with an improved sensory recovery outcome in patients who underwent digital replantation (Figure 3). The lack of clear prognostic statements for individual surgical techniques could have been caused by heterogeneity of the implemented studies concerning sensory recovery outcome and/or the inclusion of publications since 1965 into the meta-analysis—ending up with a long time range for the selected studies. From our point of view, the Highet classification system [27], which is used within this paper, classifies the sensory recovery results in a far more precise way than mean s2PD/m2PD, which is partly used in other publications. Therefore, some incomparable papers had to be excluded from this meta-analysis, which may have affected the final results of this study.

### 4.2. Influencing Factors of Nerve Regeneration

In general, it cannot be predicted in which cases a good or insufficient nerve regeneration can be expected. However, there seems to be certain factors, which influence the therapeutic success, namely, patient's age, surgeon's experience, severity and mechanism of injury, timing of repair/delay, gap length of treated nerve defect, and modern ways of medical treatment with publication date serving as indicator. By performing a meta-analysis, Ruijs et al. found out that age, delay, and type of injured nerve seemed to influence motor recovery [78]. For sensory recovery, age and delay were the significant prognostic factors [78]. The authors focused on median and ulnar injuries [78], so these results are not completely comparable to this meta-analysis. Factors affecting sensory recovery following digital nerve repair are discussed separately below.

#### 4.2.1. Age

The patient's age seems to play an important role for sensory recovery after performed replantation as proposed in 1990 by Glickman and Mackinnon [25] and confirmed by our statistical meta-analysis. Therefore, after digital replantation younger patients seem to have a better sensory recovery outcome (Figure 3). Age may affect the potential of central adaptation to peripheral nerve injury [79–81]. Nevertheless, age should not be a contraindication for digital replantation. Within this meta-analysis no correlation between patient's age and sensory recovery outcome concerning end-to-end and end-to-side coaptations, nerve grafting, artificial conduit-, vein-, and muscle-in-vein, and muscle reconstructions could be shown. In 2005, Ruijs et al. stated that youth is the main factor for a successful ulnar and median nerve repair [78]. Also, Rosén and Lundborg noted that sensory recovery benefits especially from youth [82]. Additionally, Lohmeyer et al. assessed that age is one of the major recovery predictors for patients, which were treated with end-to-end coaptations, nerve grafts and artificial conduits following nerve injuries of the upper extremity [3]. In conclusion, patient's age has to be named as one of the major predictors for nerve recovery with best results in childhood and adolescence [83].

#### 4.2.2. Surgeon's Experience

The surgeon's experience is an important factor influencing the patient's outcome [78]. Surely, there are tests and quality standards to verify the surgeon's microsurgical abilities. Nevertheless, a learning curve can be observed. In general, data sets as presented in most articles are generated from patients, who were treated by different surgeons. Unfortunately, most authors do not provide further information concerning each surgeon's personal success rate.

#### 4.2.3. Severity/Mechanism of Injury

Different types of simple or severe injuries (sharp, crush, or avulsion injuries) may significantly influence patient outcome. For a satisfying final outcome, reconstructions of tendons, vessels, bone, and skin defects have to be performed fairly often besides nerve repairs. A sharp nerve transection is easier to be treated than complex crush or avulsion injuries, and excessive postoperative scarring or mechanical stress are usually less likely [78]. Therefore, a repair of a sharp nerve injury correlates with a better patient outcome [25].

#### 4.2.4. Timing of Repair/Delay

A final statistical evaluation for the timing of repair/delay was not possible. The given data sets did not include complete coverage of all information needed. Therefore, in this meta-analysis it was not possible to state if primary or secondary repair was superior to one another. However, various publications claimed that there seems to be a better sensory recovery outcome if a nerve defect is treated primarily [25, 78]. Apart from that, there is an unfavorable prognosis for waiting more than 6 months or one year after performing a nerve repair [84–87]. For example, in some cases of strong contamination, secondary reconstruction is required. If possible, nerve continuity should be re-established primarily.

#### 4.2.5. Gap Length

Within this paper, publications with artificial conduit-, vein-, muscle-in-vein, and muscle techniques targeting gap lengths up to 4 cm were used for our statistical meta-analysis. The statistical analysis could not demonstrate that smaller gap lengths led to better sensory recovery outcomes in comparison to more extended defects if the average gap length of all publications was used for analysis. Of course, evaluation was limited since only average gap lengths were stated in most studies. On the other hand, various publications indicated that a smaller nerve defect has a better sensory recovery after certain surgical treatments [3, 84, 85, 88, 89]. Nerve regeneration also seems to deteriorate with increasing distance to the innervated organ [3]. So, if longer distances are bridged by autologous nerve grafting, recovery worsens with nerve grafts measuring more than 3 to 5 cm in length [78].

#### 4.2.6. Publication Date

More efficient techniques and broader surgical experience may have improved the overall historical clinical outcome. If publications from 1965 until 1979 were compared with those from 1980 to date, there would be a statistically significant improvement in sensory recovery outcomes after end-to-end coaptations. This represents the biggest group (1,383 nerve repairs) within this meta-analysis. Moreover, this confirms the findings by Glickman and Mackinnon [25] and can be explained by a better quality of supply during the last few decades.

#### 4.2.7. Adequate Follow-Up Period

In order to achieve feasible final results and to obtain a high level of quality, it is extremely important that an adequate follow-up period is guaranteed. From our point of view, follow-up time should be at least one year, giving the nerve enough time for regeneration. Of course, in certain cases, the physician can decide whether a shorter follow-up period is already associated with the final patient outcome. According to the literature, significant improvements of nerve regeneration after a successfully performed nerve repair can be seen at least up to a period of 3 years follow-up [78].

#### 4.2.8. Other Factors

Other factors like patient compliance [90, 91], specialized hand therapy [4, 82], cognitive capacity [92], comorbidities such as alcoholism or diabetes [93], and trauma-related psychological stress [94] may have influenced a patient's sensory recovery outcome but could not be further investigated within this meta-analysis. There also seems to be no evidence that gender influences recovery [78].

### 4.3. Valuable Comparable Publications

While this meta-analysis did not show any significant benefit for any specific technique presented, some publications with varying statistical and methodological value tried to compare individual surgical treatments. These publications often limit influencing factors and provide additional value for comparison.

End-to-end coaptation is the method of choice if tension-free nerve coaptation can be performed. For nerve injuries that cannot be repaired by direct tension-free coaptation, nerve autograft is regarded as the gold standard [95–97]. In cases where both techniques could be used for nerve repair, end-to-end coaptation should be preferred to nerve grafting, because in this instance, a second nerve coaptation, which may function as a growth barrier, can be avoided [98].

Chiu and Strauch published results showing that 2PD measurements indicated a superiority of nerve grafting and direct nerve repair in comparison to vein grafting for gap lengths of 3 cm or less [36]. For nerve defects, which were never greater than 3 cm, Laveaux et al. could demonstrate that nerve grafting was superior to vein grafting [56]. Rinker and Liau showed in a prospective randomized controlled study that digital nerve reconstructions with autologous vein grafts were comparable to the use of PGA conduits with regard to sensory recovery (gap lengths: 0.4–2.5 cm) [99]. Furthermore, the use of artificial conduits was associated with a slightly higher postoperative complication rate compared to the use of vein grafts [99]. Presumably, vein grafts are said to be effective for relatively short nerve defects (gap lengths: less than 3 cm) [100].

In a second randomized controlled study by Weber et al., the authors were not able to find a significant overall difference comparing the sensibility outcome after PGA conduit repair compared to end-to-end coaptation and nerve grafting [59]. Only subgroup analysis showed superior m2PD in nerve gaps of 4 mm or less and those of 8 mm or more for PGA conduit repair. Distinctions in s2PD were not significantly different [59]. Dahlin and Lundborg stated that artificial conduits were useful for bridging an up to 5 mm long nerve defect in human median and ulnar nerves [101]. The outcome after the use of artificial conduits in comparison to direct nerve repair was quite similar or even better [64, 101, 102]. Also, Lundborg et al. concluded that artificial conduit repair of the median and ulnar nerves seemed at least as efficient for short gap lengths (3–5 mm) as direct nerve repair [103]. Battiston et al. demonstrated that the use of muscle-in-vein grafts (gap lengths: 1.5 cm or less) and artificial conduits (gap lengths: 4 cm or less) both led to good clinical results [64]. Pereira et al. came to the conclusion that the muscle graft technique (gap lengths: 1.5–2.8 cm) is considered to be superior to the use of end-to-end coaptations [37]. 

### 4.4. Promising Upcoming Nerve Repair Techniques

Processed nerve allografts are now commercially available as Avance (AxoGen, Inc., Alachua, FL, USA). In a recent evaluation of 35 sensory allograft nerve repairs in the upper extremity, return of sensibility was found to be significant in 89% of digital nerve repairs for nerve gaps ranging from 0.5–5 cm defect length (mean 2.3 ± 1.2 mm) [23]. This recent study outcome seems considerably promising, but acceptance for allogeneic transplantation might be an issue for some patients. Further inquiry of this technique is required to estimate its role in peripheral nerve reconstruction, due to lack of the comparative useful literature, within this meta-analysis that method has not been statistically evaluated.

### 4.5. Treatment Recommendation

**Figure 4:**
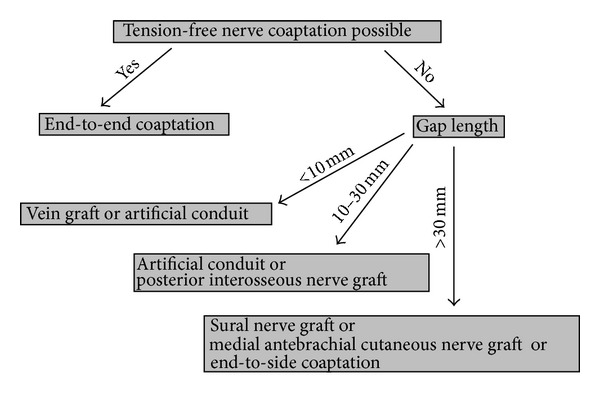
Treatment recommendation by the authors. Treatment recommendation for digital nerve repair, taking into consideration the possibility of tension-free coaptation and gap length after debridement as well as possible wound contamination, microsurgical experience, clinical setting, and patient's expectations have to be taken into regard.

Based on the findings of this meta-analysis, no clear treatment recommendations could be made. Thus, surgical decision making should be based on personal preference and clinical experience—leading up to the following treatment recommendation by the authors (Figure 4). Whenever tension-free nerve coaptation is possible, end-to-end coaptation is the method of choice for digital nerve repair. If a tension-free nerve coaptation cannot be performed, the defect length following proper debridement determines the suitability of each technique. The authors recommend performing a vein graft, or artificial conduit for gap lengths shorter than 10 mm. For gap lengths ranging from 10 to 30 mm, an artificial conduit or a posterior interosseous nerve graft should be used. For gap lengths longer than 30 mm, a sural nerve graft, a medial antebrachial cutaneous nerve graft, or end-to-side coaptation seem to be the most appropriate. The anatomical length of the posterior interosseous nerve graft ranges from 5 to 10 cm [104]. However, due to anatomical variations, we advise harvesting a posterior interosseous nerve graft only for gap lengths of 4 cm or less. The harvest of the sural nerve can cause sensibility loss at the lateral side of the foot, the harvest of the medial antebrachial cutaneous nerve may cause sensory loss at the ulnar side of the forearm, and the harvest of the posterior interosseous nerve leads to loss of proprioceptive and pain perception in the wrist. Vein grafts have the tendency to collapse [17], so we only feel comfortable using this technique for nerve defects smaller than 10 mm. Therefore, we simply recommend favoring artificial or biological conduits for short nerve defects.

## 5. Conclusion

To date, no clear advantages of a specific surgical technique for digital nerve repair could be proven. So, direct tension-free nerve repair is still the method of choice, and for extended nerve defects, different techniques seem feasible for bridging the gap. Thus, decision making has to be based upon the peculiarities of each method, surgical experience, and clinical setting including gap length, wound condition, extent of injury, and patient's demands. The predictable length of certain donor nerves and donor site morbidity must also be taken into consideration, as well as operation time and additional costs for artificial conduit grafts. However, due to a lack of useful randomized controlled studies in this scientific field, no firm final conclusions can be drawn for effectiveness of the presented surgical procedures. Therefore, more high-quality randomized controlled studies are definitely needed in order to give a conclusive statement.

## Figures and Tables

**Table 1:** The modified Highet classification.

Sensory recovery outcome	Highet	s2PD	m2PD	Recovery of sensibility
Failure	S0	—	—	No recovery of sensibility in the autonomous zone of the nerve

Poor	S1	—	—	Recovery of deep cutaneous pain sensibility with the autonomous zone of the nerve
	S1+	—	—	Recovery of superficial pain sensibility
	S2	—	—	Recovery of superficial pain and some touch sensibility
	S2+	—	—	As in S2, but with over response
	S3	>15 mm	>7 mm	Recovery of pain and touch sensibility with disappearance of over response

Good	S3+	7–15 mm	4–7 mm	As in S3, but with good localization of the stimulus and imperfect recovery of 2PD

Excellent	S4	2–6 mm	2-3 mm	Complete sensory recovery

Listings of sensory recovery outcome, the Highet classification, static two-point discrimination (s2PD), moving two-point discrimination (m2PD), and recovery of sensibility. Source: Mackinnon and Dellon [27].

**Table 2:** End-to-end coaptation.

Treatment	Author	Pub. date	Nerves with follow-up	Age (mean)	Age (range)	Follow-up time (mean)	Follow-up time (range)	Timing of repair	S0–S3 in %	S3+ in %	S4 in %	Glickman and Mackinnon [25]
End-to-end coaptation	Larsen	1958	142	—	—	—	1–7 y	All	36	64	0	+
	Weckesser	1961	24	—	7–55 y	—	—	All	24	32	44	+
	Onne	1962	8	—	<14 y	—	4–15 y	Prim.	0	0	100	+
	Onne	1962	14	—	>14 y	—	4–15 y	Prim.	57	43	0	+

Since 1965 surgical microscope												
	Buncke	1972	18	—	6–51 y	—	—	All	22	28	50	+
	Poppin	1979	62	—	6–67 y	—	5–15 y	Prim.	26	55	19	+
	Posch	1980	71	—	—	—	2–11 y	Prim.	52	48	0	+
	Young	1981	27	—	3–67 y	—	2–4 y	Prim.	10	57	33	+
	Vahvanen*	1981	18	9.5 y	1–14 y	7.5 y	2–18 y	All	0	23	77	
	Sullivan	1985	42	—	20–65 y	2 y	0.5–8.5 y	All	26	52	22	
	Berger	1988	129	—	—	2 y	—	Prim.	9	91		+
	Mailänder	1988	113	—	1–80 y	2 y	0.5–5 y	All	31	47	22	
	Chiu	1990	12	37 y	19–61 y	1.8 y	0.5–3.7 y	—	0	75	25	
	Pereira	1991	29	38 y	13–72 y	2.3 y	0.7–5.3 y	All	21	58	21	
	Altissimi**	1991	54	35 y	4–64 y	—	1–7 y	—	26	61	13	
	Chow	1993	72	—	>16 y	2 y	2 y	—	10	65	25	
	Eisenschenk	1993	204	36 y	16–68 y	7.25 y	1–15.5 y	All	54	36	10	
	Vertruyen	1994	65	23 y	<20–60 y	2.7 y	1.2–5.4 y	—	26	48	26	
	Elias	1994	83	30 y	14–70 y	—	—	—	6	64	30	
	Tadjalli	1995	37	32 y	—	2.9 y	1.3–7.25 y	—	19	32	49	
	Wang***	1996	76	—	18–79 y	—	≥1 y	All	16	37	47	
	Malizos	1997	25	37 y	19–61 y	2.3 y	1.7–4.3 y	—	8	72	20	
	Schenker	2006	5	30 y	17–51 y	1.2 y	0.75–1.5 y	—	0	60	40	
	Sommer	2009	53	42 y	15–85 y	4.2 y	1.3–7.3 y	All	18	40	42	

Listings of treatment, author, publication date (pub. date), nerves with follow-up, age (mean and range; y: year), follow-up time (mean and range), timing of repair (all: primary and secondary; prim.: primary), sensory recovery (S0–3, S3+, and S4), and Glickman and Mackinnon [25] (+: publication already mentioned in Glickman and Mackinnon [25]); ∗: children ≤14 y/not 100% conform with the Highet classification/3–5 mm: 14 patients/6–15 mm: 4 patients/16–25 mm: 0 patients/patients with S4 could be less; ∗∗: not 100% conform with the Highet classification/≤5 mm: 7 patients/6–10 mm: 20 patients/11–15 mm: 13 patients/>15 mm: 14 patients/patients with S4 could be less; ∗∗∗: not 100% conform with the Highet classification/≤7 mm: 36 patients/8–15 mm: 28 patients/>15 mm: 12 patients/patients with S4 could be more.

**Table 3:** End-to-side coaptation.

Treatment	Author	Pub. date	Nerves with follow-up	Age (mean)	Age (range)	Follow-up time (mean)	Follow-up time (range)	Timing of repair	S0–S3 in %	S3+ in %	S4 in %	Nerve coaptation with epineural window
End-to-side coaptation	Pélissier	2001	6	33 y	13–46 y	1 y	0.5–1.25 y	—	17	83	0	In only some cases
	Frey	2003	2	27 y	12–42 y	3.6 y	3.1–4 y	—	0	0	100	In all cases
	Voche	2005	11	30 y	9–50 y	1.3 y	0.75–2.4 y	—	0	91	9	In all cases
	Landwehrs	2008	5	52 y	42–59 y	1.75 y	0.9–3.25 y	—	20	40	40	In all cases
	Artiaco*	2010	7	45 y	20–62 y	3 y	0.7–5 y	All	14	86	0	In all cases

Listings of treatment, author, publication date (pub. date), nerves with follow-up, age (mean and range; y: year), follow-up time (mean and range), timing of repair (all: primary and secondary), sensory recovery (S0–3, S3+, and S4), and nerve coaptation with epineural window; ∗: only original data set by Artiaco is shown; the other data sets (Pélissier, Frey, and Voche) are demonstrated separately.

**Table 4:** Nerve graft.

Treatment	Author	Pub. date	Nerves with follow-up	Age (mean)	Age (range)	Follow-up time (mean)	Follow-up time (range)	Timing of repair	Gap length (mean)	Gap length (range)	S0–S3 in %	S3+ in %	S4 in %	Glickman and Mackinnon [25]
Nerve graft	Seddon	1947	15	—	>18 y	—	2–4 y	—	—	3–8 cm	82	6	12	+

Since 1965 surgical microscope														
	McFarlane	1976	13	38 y	20–59 y	1.2 y	0.6–1.9 y	—	—	1.5–3.5 cm	54	46	0	+
	Wilgis	1979	11	—	17–54	—	0.9–2 y	—	—	1–2.5 cm	0	33	67	+
	Young	1979	27	27 y	15–57 y	—	0.5–5 y	—	—	>2 cm	60	25	15	+
	Yamano	1982	5	44 y	28–56 y	2.2 y	2–2.5 y	Sec.	3.4 cm	2–5 cm	0	100	0	+
	Tenny***	1984	42	28 y	9–59 y	2.5 y	0.5–5.7 y	—	2 cm	0.5–6 cm	24	36	40	
	Beazley	1984	12	—	0.5–71 y	—	2–6 y	—	—	1–5 cm	42	58	0	+
	Rose	1985	5	—	>18 y	—	3–7 y	—	—	5–8 cm	20	20	60	+
	Rose	1989	13	—	19–55 y	1 y	—	—	4.5 cm	—	7	55	38	+
	Nunley	1989	21	29 y	16–51 y	4.7 y	2–7.4 y	All	2.5 y	1.5–4 cm	14	57	29	
	Chiu	1990	4	37 y	19–61 y	2 y	1.2–2.75 y	All	2.7 cm	1.8–3 cm	0	100	0	
	Mackinnon	1990	31	—	18–62 y	6 y	—	—	—	1–5 cm	6	40	54	+
	Dumontier	1990	14	27 y	7–53 y	3.6 y	1–7.7 y	All	2.5 cm	1.5–3 cm	79	14	7	
	Eisenschenk	1993	96	36 y	16–68 y	7.25 y	1–15.5 y	All	2.2 cm	1–6 cm	59	29	12	
	Kracht	1993	22	—	2–66 y	2.8 y	1–5 y	All	—	—	64	36	0	
	Wang*	1996	14	—	18–79	—	≥1 y	All	3 cm	1.5–6 cm	14	36	50	
	Inoue**	2002	3	26 y	18–31 y	0.8 y	0.5–1 y	All	1.3 cm	1–1.5 cm	0	0	100	
	Schonauer^∗∗ ∗∗∗^	2008	8	23 y	23 y	1 y	1 y	—	—	—	0	100	0	
	Chiu**	2009	1	17 y	17 y	0.5 y	0.5 y	Sec.	2 cm	2 cm	0	0	100	
	Karabekmez	2009	8	44 y	23–65 y	0.75 y	0.4–1 y	—	2.2 cm	0.5–3 cm	0	37	63	
	Sommer	2009	4	27 y	13–68 y	4.2 y	1.3–7.3 y	All	—	—	0	0	100	
	Laveaux	2010	15	33 y	13–56 y	16.8 y	11.6–23.7 y	—	—	≤3 cm	20	67	13	

Listings of treatment, author, publication date (pub. date), nerves with follow-up, age (mean and range; y: year), follow-up time (mean and range), timing of repair (all: primary and secondary; sec.: secondary), gap length (mean and range), sensory recovery (S0–3, S3+, and S4), and Glickman and Mackinnon [25] (+: publication already mentioned in Glickman and Mackinnon [25]); ∗: not 100% conform with the Highet classification/≤7 mm: 7 patients/8–15 mm: 2 patients/>15 mm: 5 patients/patients with S4 could be more; ∗∗: case report; ∗∗∗: Y-shaped nerve grafts.

**Table 5:** Artificial conduit.

Treatment	Author	Pub. date	Nerves with follow-up	Age (mean)	Age (range)	Follow-up time (mean)	Follow-up time (range)	Timing of repair	Gap length (mean)	Gap length (range)	S0–S3 in %	S3+ in %	S4 in %	Conduit material
Artificial conduit	Mackinnon	1990	15	30.5 y	23–38 y	1.9 y	0.9–2.7 y	Sec.	1.7 cm	0.5–3 cm	13	54	33	Polyglycolic acid
	Weber	2000	46	36 ± 14 y	17–65 y	0.8 ± 0.37 y	0.25–1 y	All	0.7 ± 0.56 cm	0–3 cm	26	30	44	Polyglycolic acid
	Inada*	2004	1	62 y	62 y	0.3 y	0.3 y	Sec.	2 cm	2 cm	0	0	100	Polyglycolic acid/collagen
	Battiston	2005	19	40 y	15–67 y	2.5 y	0.5–6.2 y	All	2 cm	1–4 cm	31	58	11	Polyglycolic acid
	Dellon*	2006	2	42 y	42 y	2.5 y	2.5 y	Sec.	3 cm	3 cm	0	0	100	Polyglycolic acid
	Bushnell	2008	9	35 y	18–50 y	1.25 y	1–1.8 y	All	—	1-2 cm	0	56	44	Collagen
	Lohmeyer	2009	12	38 y	12–66 y	1 y	0.25–1 y	All	1.25 cm	0.8–1.8 cm	25	42	33	Collagen
	Thomsen	2010	11	30 y	16–49 y	1 y	0.5–1.4 y	Sec.	1.1 cm	0.5–2 cm	8	55	37	Collagen

Listings of treatment, author, publication date (pub. date), nerves with follow-up, age (mean and range; y: year), follow-up time (mean and range), timing of repair (all: primary and secondary; sec.: secondary), gap length (mean and range), sensory recovery (S0–3, S3+, and S4), and conduit material; ∗: case report.

**Table 6:** Vein graft.

Treatment	Author	Pub. date	Nerves with follow-up	Age (mean)	Age (range)	Follow-up time (mean)	Follow-up time (range)	Timing of repair	Gap length (mean)	Gap length (range)	S0–S3 in %	S3+ in %	S4 in %	Conduit material
Vein graft	Walton*	1989	18	32 y	15–55 y	1.2 y	0.7–2 y	—	1.4 cm	1–3 cm	39	50	11	Vein
	Chiu	1990	10	37 y	19–61 y	3.2 y	1.3–6 y	All	2.6 cm	1–3 cm	20	80	0	Vein
	Tang	1995	9	26 y	17–46 y	3 y	2.5–3.5 y	—	3.1 cm	2–5.8 cm	22	78	0	Vein
	Malizos	1997	23	37 y	19–61 y	2.6 y	1.6–5 y	—	1.7 cm	1.2–2.8 cm	4	74	22	Vein
	Risitano	2002	22	35 y	15–70 y	1.75 y	0.5–5 y	—	1.5 cm	0.5–2 cm	50	0	50	Vein
	Lee	2008	3	32 y	19–52 y	8.6 y	2.75–12 y	Sec.	1.4 cm	0.8–2.5 cm	33	67	0	Vein
	Laveaux	2010	17	46 y	27–75 y	5.2 y	0.9–8.8 y	—	—	≤3 cm	35	65	0	Vein

Listings of treatment, author, publication date (pub. date), nerves with follow-up, age (mean and range; y: year), follow-up time (mean and range), timing of repair (all: primary and secondary; sec.: secondary), gap length (mean and range), sensory recovery (S0–3, S3+, and S4), and conduit material; ∗: m2PD and not s2PD used for classification.

**Table 7:** Other techniques (muscle graft, muscle-in-vein graft, and digital artery graft).

Treatment	Author	Pub. date	Nerves with follow-up	Age (mean)	Age (range)	Follow-up time (mean)	Follow-up time (range)	Timing of repair	Gap length (mean)	Gap length (range)	S0–S3 in %	S3+ in %	S4 in %	Conduit material
Other techniques	Norris	1988	7	42 y	16–61 y	0.6 y	0.25–0.9 y	—	—	1.5–2.5 cm	28	57	15	Muscle
	Pereira	1991	12	37 y	18–64 y	2.1 y	0.5–3.3 y	All	—	1.5–2.8 cm	0	17	83	Muscle
	Calder	1993	2	—	15–17 y	—	0.8 y	All	—	2–6 cm	0	50	50	Muscle
	Battiston	2005	13	36 y	20–50 y	3.1 y	1.7–5 y	All	1.1 cm	0.5–1.5 cm	24	38	38	Muscle-in-vein
	Kosutic*	2009	2	—	—	2 y	2 y	Prim.	2.6 cm	2-3 cm	0	50	50	Digital artery
	Marcoccio	2010	22	38 y	11–61 y	3.6 y	1.5–8 y	—	2.2 cm	1–3.5 cm	60	26	14	Muscle-in-vein

Listings of treatment, author, publication date (pub. date), nerves with follow-up, age (mean and range; y: year), follow-up time (mean and range), timing of repair (all: primary and secondary; prim.: primary), gap length (mean and range), sensory recovery (S0–3, S3+, and S4), and conduit material; ∗: case report.

**Table 8:** Finger and thumb replantation.

Treatment	Author	Pub. date	Nerves with follow-up	Age (mean)	Age (range)	Follow-up time (mean)	Follow-up time (range)	S0–S3 in %	S3+ in %	S4 in %	Glickman and Mackinnon [25]	Replanted digits
Replantation	Chow	1977	10	—	—	1.5 y	—	0	100		+	Thumb
	Gelberman	1978	35	—	4–47 y	—	—	48	26	26	+	Thumb
	Schlenker	1980	25	31 y	—	—	0.5–3 y	44	56		+	Thumb
	May	1982	23	21.2 y	—	2.5 y	—	9	78	13	+	Thumb
	Yamauchi	1982	186	—	1.2–68 y**	—	>0.5 y	37	39	24		Finger
	Yoshimura*	1982	365	—	1–68 y	—	>0.5 y	32	44	24		All
	Yamano	1985	74	33.5 y	—	—	—	4	96		+	All
	Nylander	1987	5	37.4 y	—	1.7 y	—	20	60	20	+	Finger
	Nylander	1987	3	19.3 y	—	1.7 y	—	0	100		+	Thumb
	Blomgren	1988	33	39 y	—	2.25 y	—	88	6	6	+	All
	Goldner	1989	24	23 y	—	5 y	—	5	95		+	All
	Ikeda***	1990	14	4 y	1.2–9 y	8 y	3–14 y	0	7	93		All
	Ahcan	1997	22	42 y	21–58 y	4.7 y	2–7 y	32	50	18		Finger
	Dos Remédios	2005	46	36.5 y	13–63 y	—	>1 y	19	59	22		Finger
	Walaszek	2008	59	38 y	11–74 y	3.5 y	1–6 y	20	75	5		All

Listings of treatment, author, publication date (pub. date), nerves with follow-up, age (mean and range; y: year), follow-up time (mean and range), sensory recovery (S0–3, S3+, and S4), Glickman and Mackinnon [25] (+: publication already mentioned in Glickman and Mackinnon [25]), and replanted digits (details on which digit got replanted); ∗: not 100% conform with the Highet classification/≤5 mm: 87 patients/6–10 mm: 92 patients/11–15 mm: 69 patients/16–20 mm: 49 patients/>20 mm 68 patients/patients with S4 could be less; ∗∗: patients with age of 20–60 made up 78% of the whole group; ∗∗∗: m2PD and not s2PD used for classification.

**Table 9:** Pooled estimates.

	S0–3		S3+		S4	
	Number of studies pooled	Pooled proportion, % (95% CI)	Number of studies pooled	Pooled proportion, % (95% CI)	Number of studies pooled	Pooled proportion, % (95% CI)
End-to-end coaptation	20	21 (12–27)	19	50 (44–56)	19	28 (20–36)
End-to-side coaptation	5	12 (3–25)	5	67 (40–89)	5	21 (3–50)
Nerve graft	18	27 (16–40)	18	42 (33–52)	18	25 (14–37)
Artificial conduit	6	20 (12–30)	6	45 (35–56)	6	34 (23–46)
Vein graft	6	30 (15–48)	6	51 (20–82)	6	14 (3–32)
Muscle graft	3	11 (0–36)	3	37 (13–65)	3	51 (10–91)
Muscle-in-vein graft	2	42 (13–75)	2	32 (18–48)	2	25 (6–51)
Replantation	15	24 (14–35)	10	43 (31–56)	10	23 (15–32)

Pooled estimates of proportions for S0–3, S3+, and S4 with 95% confidence intervals from the random effects model presenting sensory recovery outcomes after end-to-end and end-to-side coaptations, nerve grafts, artificial conduit-, vein-, muscle, and muscle-in-vein reconstructions, and replantations.
